# Development of Mobile Laboratory for Viral Hemorrhagic Fever Detection in Africa

**DOI:** 10.1093/infdis/jiy362

**Published:** 2018-06-15

**Authors:** Manfred Weidmann, Ousmane Faye, Oumar Faye, Ahmed Abd El Wahed, Pranav Patel, Christophe Batejat, Jean Claude Manugerra, Aimee Adjami, Matthias Niedrig, Frank T Hufert, Amadou A Sall

**Affiliations:** 1 Institute of Aquaculture, University of Stirling, Scotland, United Kingdom; 2 Arbovirus Unit, Pasteur Institute, Dakar, Senegal; 3 Unit of Infection Models, German Primate Center, Goettingen, Germany; 4 Division of Microbiology and Animal Hygiene, University of Goettingen, Germany; 5 TIB Molbiol Syntheselabor GmbH, Berlin, Germany; 6 Laboratory for Urgent Response to Biological Threats (CIBU), Environment and Infectious Risks Unit, Institut Pasteur, Paris, France; 7 Multi Disease Surveillance Centre WHO, Ougadougou, Burkina Faso; 8 Robert Koch Institute, Berlin, Germany; 9 Institute of Microbiology and Virology, Brandenburg Medical School Fontane (and Member of the Faculty of Environment and Natural Sciences of B-TU Senftenberg site), Senftenberg, Brandenburg, Germany

**Keywords:** Ebola virus disease, laboratory suitcase, mobile laboratory, point of care, viral hemorragic fever virus

## Abstract

**Background:**

A mobile laboratory transportable on commercial flights was developed to enable local response to viral hemorrhagic fever outbreaks.

**Methods:**

The development progressed from use of mobile real-time reverse-transcription polymerase chain reaction to mobile real-time recombinase polymerase amplification. In this study, we describe various stages of the mobile laboratory development.

**Results:**

A brief overview of mobile laboratory deployments, which culminated in the first on-site detection of Ebola virus disease (EVD) in March 2014, and their successful use in a campaign to roll back EVD cases in Conakry in the West Africa Ebola virus outbreak are described.

**Conclusions:**

The developed mobile laboratory successfully enabled local teams to perform rapid disgnostic testing for viral hemorrhagic fever.

## Teaser

We developed a mobile laboratory for molecular detection of viral hemorrhagic fever viruses for an African team that tested its function during several outbreaks. This was the first team deployed onsite during the Ebola outbreak in West Africa 2014–2016.

Timely viral hemorrhagic fever (VHF) diagnostics in Africa are difficult due to distances. Not many specialized laboratories are available, and by the time samples reach the National Institute for Communicable Diseases (Johannesburg, South Africa) or the World Health Organization (WHO) Reference Center for Yellow Fever at the Institut Pasteur de Dakar (IPD), they are often not fit for use.

Time to result can be very long. Many outbreaks in the past decades have shown that the initial lag phase of the outbreaks regularly cause infections in healthcare workers. Thus, VHF outbreaks amplify and quickly grow out of proportion before control measures are initiated, as the recent huge outbreak of Ebola virus disease (EVD) in West Africa has shown.

Therefore, the weak link is the lack of mobile onsite diagnostics. Due to the rare occurrence of these diseases (EVD cases: 2500 [1976–2013], 28 639 [2014–2016]; West Africa [1]), commercial interest is very low, and only a few kits usually confectioned for use in standard laboratories are available [2]. The challenge is to take the laboratory into the field for early detection and response in simple infrastructure settings independent of electricity supply and cool chain.

Viral hemorrhagic fevers are infectious febrile diseases that can develop into generalized hemorrhages. Manifestations are unspecific and show great variability usually with early fever, hypotension, bradycardia, tachypnoea, conjunctivitis, pharyngitis, and sometimes quite variable exanthema, late hemorrhagic diathesis, mucosal or cunjunctival hemorrhages, hematuria, hematemesis, melena, intravascular coagulation, and shock. Central nervous system manifestations include convulsions, delirium, or coma, all of which are predictors of mortal outcome [3, 4]. Viruses eliciting these diseases in Africa belong to 4, single-strand, ribonucleic acid (RNA) virus families: (1) *Arenaviridae* (Lassa virus [LAV] and Lujo virus [LUJV]); (2) *Filoviridae* (Marburg virus [MARV], Sudan virus [SUDV], Ebola virus [EBOV], and Bundibugyo virus [BDBV]); (3) *Bunyaviridae* (Rift Valley fever virus [RVFV] and Crimean-Congo hemorrhagic fever virus [CCHFV]); (4) *Flaviviridae* (yellow fever virus [YFV] and dengue virus [DENV]). Lujo virus and a new Rhabdovirus, Bas-Congo virus (BASV), were only recently described and have not yet caused epidemics [5, 6].

With the exception of RVF, dengue fever (DF), and YF, VHF can be transmitted by direct contact with blood or body fluids of infected patients, facilitating nosocomial infections. Incubation times range from 2 to 21 days. Because of the high morbidity and mortality caused by VHF, outbreaks elicit huge media attention driven by the fear of cases exported to industrialized countries, which sporadically occur. There is minimal clinical and epidemiological knowledge on VHF, and, as the outbreak in West Africa showed, the medical infrastructure is bad or almost nonexistent, which amplifies these outbreaks.

A general case definition for VHF is severe disease with vascular symptoms in a patient who lives or stayed in a VHF-endemic area or who had contact with live or dead reservoir animals (blood, excrements, urine) and/or VHF patients and their bodily fluids.

In the field, diagnostic antibody (immunoglobulin [Ig]M, IgG) detection assays (enzyme-linked immunosorbent assay [ELISA], immunofluorescence assay) developed by specialized laboratories need at least a tent laboratory, as recently shown in the West African EVD outbreak [7]. Although these are not required for diagnosis of acute disease, they are important to confirm resolved disease or to monitor disease that has progressed beyond the diagnostic window for molecular detection [8].

Virus detection methods include virus isolation or capture ELISAs, requiring cell culture or derived antigen preparations produced in biosafety level-3 or -4 laboratories, with the exception of recombinant antigens. Some rapid lateral flow assay formats with varying degrees of sensitivity and specificity have been described for EBOV detection [9, 10]. The direct detection of the virus genome by quantitative real-time reverse-transcription polymerase chain reaction qRT-PCR has proven to be successful in the large ongoing CCHF epidemic in Turkey and in the recent EVD outbreak in West Africa [11–15].

We set out to develop mobile molecular detection for hemorrhagic fever viruses (HFVs) with a vision to train a local team to deploy and perform VHF diagnostics in outbreak situations. The main characteristics of the laboratory were mobility (on commercial flights) and independence of electricity and cool chain. We describe the development of the mobile laboratory (ML) initially using qRT-PCR and later real-time recombinase polymerase amplification (RT-RPA) through 4 stages (ML1–4) and discuss successful deployments to Cape Verde, Mauritania, Uganda, and finally Guinea.

## MATERIALS AND METHODS

### Mobility

In ML1–2 stages, for qRT-PCR, we chose the SmartCycler (Cepheid, Sunnyvale, CA) [16, 17], which was also used by other teams [18–21]. In ML3–4a/b stages, for RT-RPA, we used the Twista (TwistDx, Cambridge, UK) and the TS2.2 device (QIAGEN Lake Constance, Stockach, Germany).

### Electricity Supply

In ML1–3, electricity was tapped from a motor vehicle battery via inverter (HPL 1200-D-12 inverter, 12V, 1200W). In ML3, electricity was tapped via solar panel and power pack Yeti 1250. Finally, in ML4, electricity was tapped via solar panel and power pack Yeti 400 (GOALZERO, Bluffdale, UT) (Table 1).

**Table 1.** Mobile Laboratory Components for ML1–4a/b

Mobile Laboratory	Items	ML1	ML2	ML3	ML4a	ML4b
Electricity supply	Converter	1	1	1		
	Solar power pack			1	1	1
Extraction	Centrifuge	MiniSpin				
	Rotator		Intelli-Mixer	Intelli-Mixer		
	Heating block				1	1
	Pipettes	2	1	1	1	1
Amplification	Detection device	Smart Cycler	Smart Cycler	Twista	Twista	TS2.2
	Laptop	Laptop	Laptop	Laptop	Laptop	----
	Centrifuge	SC spin down	SC spin down	Myfuge	Spin down	Spin down
	Lab dancer	1	1	2	2	2
	Pipettes	6	5	5	2	2
Energy demand		522W	464W	467W	196W	173W
Biochemistry	Extraction	QIAGEN Mini Kit	QIAGEN Mini Kit/ Dynabeads SILANE Viral NA	innuPREP MP Basic Kit	QIAGEN SpeedXtract kit	QIAGEN SpeedXtract kit
	Oligonucleotides	Individual	Dried 4× mixes 10× mixes positive controls	Dried 40× mixes positive control	Dried positive control	Dried positive control
	Amplification kit	QIAGEN QuantiTect Probe RT-PCR Kit	QIAGEN QuantiTect Probe RT-PCR Kit	Twist Exo RT-RPA Kit	Twist Primer-in pellets	Twist Primer-in pellets

Abbreviations: lab, laboratory; ML, mobile laboratory; RT-PCR, reverse-transcription polymerase chain reaction; RT-RPA, real-time recombinase polymerase amplification.

### Cold Chain Independence

**Figure 1.**
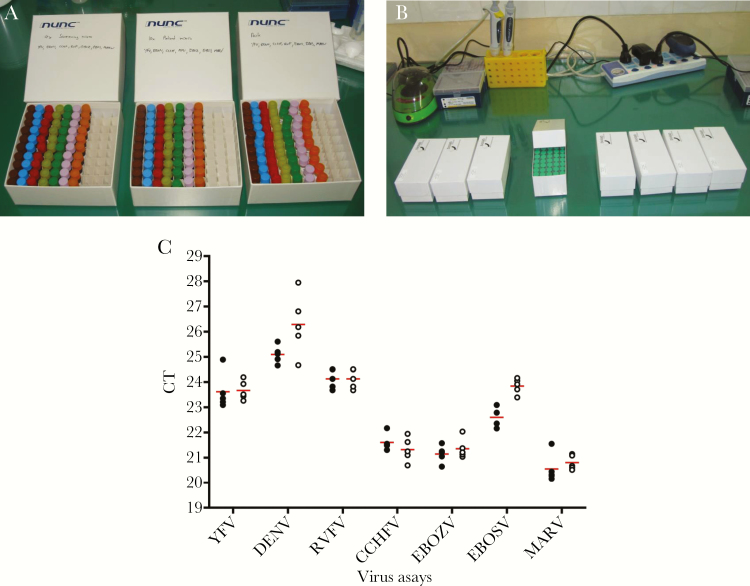
Dried primer and probe mixes. (A) Quantitative reverse transcription-polymerase chain reaction (qPCR) mixes: box 1, 4× screening mix; box 2, 10× PCR mix; box 3, ribonucleic acid (RNA)-positive controls. Tubes in brown: yellow fever virus (YFV), blue; dengue virus (DENV), red; Crimean-Congo hemorrhagic fever virus (CCHFV), light-green; Rift Valley fever virus (RVFV), green; Ebola virus (EBOV), pink; Sudan virus (SUDV), orange; Marburg virus (MARV). (B) Real-time recombinase polymerase amplification (RT-RPA) 40× mixes (50 per box): box 1, YFV; box 2, DENV1–3; box 3, DENV 4; box 4, Bundibugyo virus (BDBV); box 5, SUDV; box 6, EBOV; box 7, MARV1; box 8, MARV2. (C) Results of testing all hemorrhagic fever (VHF) mixes (n = 5) in Kedougou (black circles) and in Dakar (white circles); mean is depicted in red.

**Figure 2.**
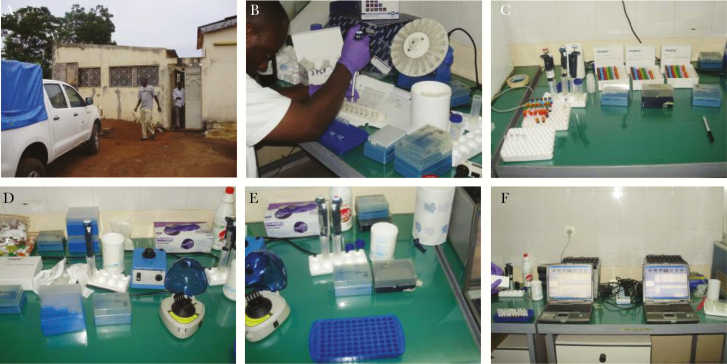
Mobile laboratory (ML)1–3 pipetting. (A) Field station in Kedougou: (B) site 1, extraction; (C) site 2, master mix; (D) site 3, sample meets mix; (E) site 4, positive control meets mix; (F) site 5, amplification devices. The ML3 shown in [23].

Initially, the QuantiTect Probe RT-PCR Kit (QIAGEN, Hilden, Germany) was taken in a cooling bag and kept at 4°C in a refrigerator onsite because refrigerators are available in most places, even in remote Africa. This kit could be used for up to 2 weeks under these conditions, which was usually used up by that time. The ML1 used individual primers and probes. The ML2 used 3 custom-made dried mixes containing primers and probe (Roboscreen, Jena, Germany). A 4× screening mix (1 sample, 1 positive and 1 negative control, 1 volume for pipetting safety, and 40 pmol primers/20 pmol probe) was used. The tubes were arranged in one box (Figure 1A, box 1) to use them at an outbreak site to identify any of 7 HFVs. The second box contained 10× mixes (100 pmol primers/50 pmol probe), allowing us to test 7 samples, for continuous screening of samples (Figure 1A, box 2). The third box contained dried synthetic RNA-positive controls, into which 1 volume was transferred from either the 4× or 10× tube for the positive control reaction (Figure 1A, box 3). The ML3 was cool chain independent: RT-RPA were reagents supplied as dried pellets in 8-strip tubes sealed in aluminium foil pouches. The dried primers and probe concept was adapted by using 40× tubes (840 pmol of each primer/400 pmol probe (TIBMOLBIOL, Berlin, Germany). One 40× stock solution resolved in 200 µL water (4.2 µM of each primer/2 µM probe) yielded 4 9× master mixes for 8-tube strips by transferring 45 µL into each 9× master mix (Figure 2B, Table 2). The ML4 used a bespoke TwistAmp exo RT kit (TwistDx, Cambridge, United Kingdom) with dried pellets already containing the primers and probe. Each pellet contained 21 pmol of each primer and 6 pmol probe and MgCl_2_ (TwistDx, Baraham, United Kingdom [22]).

**Table 2.** List of RT-PCR and RT-RPA Oligonucleotides

Virus	Target Gene	Primer and Probe Sequences	
qRT-PCR			
CCHFV	N-gene	CCS FP	GG**Y**AC**Y**AAGAAAATGAAGAAGG
		CCS P	CTGAGCAC**H**CCAATGAA**R**TGGGG
		CCS RP	CRGGGA**K**TGT**Y**CC**R**AAGCA
BDBV	N-gene	BDB FP	AGGATGGAAACCAAGGCGA
		BDB RP	TCATGATTTTCGGATCTGTCCTG
		BDB P	CAACCAATACAGAGACAAGCCAATGCCAC
SIGV	G-gene	SIG FP	GTGACATTCCAAGTAACTGATT
		SIG RP	CAACGGCAGTTTGGATA
		SIG P1	CCCTCCGTGTCCTCCCGGTACC
RT-RPA			
BDBV	N-gene	BDB RPA FP3	AAGCTGAGAAATGGACAGGACCAGGATG
		BDB RPA RP1	CTGGACTGTGTTTGAAGGGTTTGGTCATG
		BDB RPA P	ATCTGTCCTGTACTTGTGGCATTGGCTT-BTF-TCTGTATTGGTTG-P

Degenerated nucleotides in bold (IUB code).

Abbreviations: BDBV, Bundibugyo virus; BUD RPA-P BTF, B, thymidine nucleotide carrying Blackhole quencher 1; CCHFV, Crimean-Congo hemorrhagic fever virus; F, thymidine nucleotide carrying Fluorescein; P, phosphate: 3’phosbate to block elongation; qRT-PCR, quantitative reverse-transcription polymerase chain reaction polymerase chain reaction; RT-RPA, real-time recombinase polymerase amplification; SIGV, Sigma virus; T, tetrahydrofuran spacer.

### Amplification

**Figure 3.**
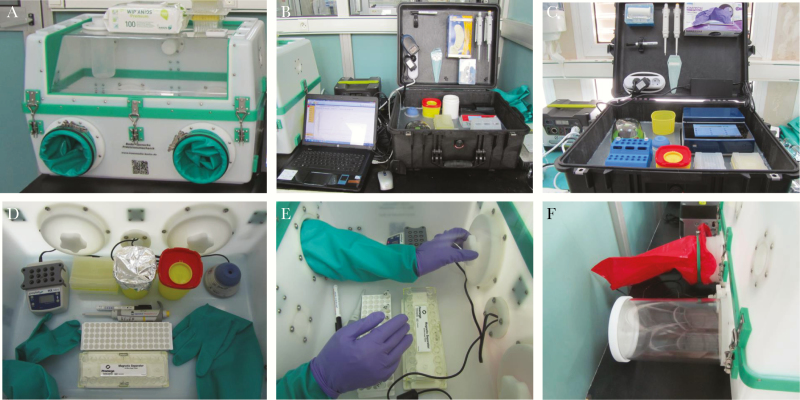
Mobile laboratory (ML)4 pipetting. (A) Extraction in glovebox. (B) ML4a Real-time recombinase polymerase amplification (RT-RPA) in development of a suitcase laboratory (DiaS). (C) The ML4b and 2 RT-RPA in DiaS (Tubescanner II, no laptop). (D) Details of setup for extraction. (E) Opening of internal door of export lock. (F) Export lock and waste bag attachment.

ML1–3 were organized into 5 sites in close proximity (Figure 2B–F, [23]). ML4 had only 2 sites (Figure 3A and B [24]). Quantitative RT-PCR was performed using published primers and probes and RNA standards for RVFV [25], YFV [26], EBOV, SUDV, MARV [27, 28], and DENV [29]. For CCHFV, the qRT-PCR design was in reference to published sequences from African CCHFV isolates (NC_005302, U88410, EF123122, U88411, U88415, U88416); for BDBV, primers were in reference to sequences FJ217161 and NC_014373 (Table 2). Quantitative RNA standards for CCHFV and BDBV were transcribed from the CCHFV-S-segment, and BDBV-N-gene was ligated into pCRII and evaluated as previously described [28]. The qRT-PCR temperature profile was as follows: room temperature 50°C/5 minutes, activation 95°C/15 minutes, and 45 cycles of 95°C/5 seconds and 60°C/15 seconds. For CCHFV, the same profile was used with a touchdown in 2-degree steps from 70°C to 64°C for 3 cycles each and 33 cycles at 62°C was used.

The Sigma virus (SIGV) qRT-PCR (sensitivity, 10 RNA molecules detected) used for the evaluation of extraction kits was performed using the LightCycler 480 RNA Master Hydrolysis Probes kit (Roche, Mannheim, Germany) and temperature profile: room temperature 63°C/3 minutes, activation 95°C/30 seconds, and 45 cycles of PCR at 95°C/5 seconds and 53°C/15 seconds, followed by cooling at 40°C/30 seconds. Real-time RPA was performed by using the TwistAmp exo RT kit and dried oligonucleotides as previously described [30]. In ML4, the bespoke TwistAmp exo RT kit was used with 45 μL rehydration solution, and 5 µL RNA template was added to each pellet containing tube. The tubes were mixed, centrifuged, and placed in the Twista for real-time monitoring of fluorescence. Reaction was performed at 42°C for 20 minutes, as previously described [22].

### Extraction

Six commercially available extraction kits were tested in reference to the QIAGEN QIAamp viral RNA kit ([1] QIAGEN QIAamp MinElute Virus Spin; [2] Roche HighPure Viral Nucleic Acid; [3] Macherey-Nagel NucleoSpin RNA Virus; [4] Invitrogen PureLink Viral RNA/deoxyribonucleic acid (DNA) Mini; [5] Invitek RTP DNA/RNA virus Mini; [6] Invitrogen Dynabeads SILANE Viral NA). Using Schneider S2-cells and concentration of virus culture supernatant through ultracentrifugation columns (Amicon Ultra-15; Millipore, Darmstadt, Germany), a concentration of 1.7 × 10^12^/mL SIGV RNA molecules was obtained, as confirmed by qRT-PCR.

For the evaluation of the extraction efficiency, 40 μL SIGV stock suspension added to 200 µL serum was diluted 1:5 in molecular grade H_2_O, and 140 µL was used for extraction. The required sample volume for each kit sample was adjusted with H_2_O. Each extraction was performed in duplicate, and the extracts were tested and quantified by qRT-PCR. To simulate field conditions, all of the kits, reagents, and materials were held at 36°C for 1 day, and the experiments were repeated in an incubation room at 36°C with a relative humidity of 23%–27%. SpeedXtract kit evaluation was described elsewhere [22]. A hard plastic glovebox consisting of 2 halves, which can be clicked together, was purchased from Bodo Könnecke Präzisonsmechanik (Berlin, Germany). It features one shunt at the top to enter samples, 1 double-doored shunt for export of extracts, and 1 shunt to attach a double waste bag (Figure 2A and D–F).

## RESULTS

### Choice of Extraction Kits

Of the 6 commercial extraction kits tested in comparison with the QIAGEN QIAamp viral RNA kit, only kit nos. 3 (column based) and 6 (magnetic bead based) performed as well as the reference kit. We performed an additional assessment of the selected extraction methods under field conditions at 37°C ambient temperature and 25% humidity. The quantity of recovered nucleic acid was comparable with the default settings at 25°C (Table 3). Using a magnetic bead stand, the field team preferred to use kit no. 6 instead of a centrifuge for extraction. For ML4a/b, this extraction method was replaced by the SpeedXtract kit (QIAGEN Lake Constance [22]).

**Table 3.** Comparative Extraction Efficiency of Final Extraction Kit Selection

Sample Type	Absolute Extraction Result			Efficiency Ratio (Tested Kit/Reference Kit)	
	Reference Kit	Kit No. 4	Kit No. 7	Kit No. 4	Kit No. 7
SIGV supernatant	1.6 × 10^8^	6.4 × 10^7^	1.4 × 10^8^	0.40	0.9
SIGV spiked serum	9.0 × 10^7^	4.1 × 10^7^	9.2 × 10^7^	0.46	1.0

Abbreviations: SIGV, Sigma virus.

### Field Trials

In 2010, in a field trial, ML2 was tested in the field laboratory of IPD in Kedougou, Senegal (Figure 2A). All dried qRT-PCR mixes were tested using the dried RNA standards. Five assays for each RNA standard yielded an intra-assay variability from 0.32 to 0.72 cycle threshold (CT), which was confirmed when returning to the laboratory in Dakar (intra-assay variability 0.28–1.21 CT), and an overall interassay variability from 0.36 to 1.05 CT (Figure 1C).

### Deployments

All deployments of the ML from 2009 to 2014 were upon request of the WHO and/or Ministry of Health (MoH) of the different countries affected by arbovirus and VHF outbreaks. Backup serological diagnostics at IPD used in-house IgM/IgG ELISA assays for RVFV, DENV, and YFV. It also provided partial sequencing of DENV E- [31], NS5- [32], and NS5/3-NCR [33] regions and of RVFV L, M, and S segments [34].

### Dengue Virus 3 Outbreak, 2009, Cape Verde Islands

In October 2009, the WHO Collaborating Centre for Reference research on arboviruses of the IPD diagnosed the first DF cases from 46 human sera sent by the Division of Epidemiological Surveillance, MoH of Cape Verde. The latter, in collaboration with WHO, then invited the IPD to support investigation, management, and response to the epidemic, and a team of entomologists and virologists visited Cape Verde from October 26 to November 27, 2009. An in-house IgM/IgG ELISA assay, a classic PCR protocol [35] and ML1, were deployed at the blood bank of the Hospital Agostinho Neto in Praia, to test suspect DF cases. Of 496 sera analysed in total, 1 cohort of samples (52 of 189; 27%) tested positive by qRT-PCR, and another chohort (182 of 399; 46%) tested positive by classic RT-PCR. Dengue virus was detected on 6 of 10 islands. The island of Santiago was the most affected by the DF epidemic with 213 of 439 positive samples, in comparison to Sal (8 of 19), Fogo (3 of 9), Brava (3 of 8), Sao Vicente (2 of 7), and Maio (3 of 6).

Blood bank sera were tested to avoid probable transfusion of the virus from asymptomatic blood donors [36]. Twenty-five samples tested negative for DENV by qRT-PCR, and 4 of 14 tested positive by nested RT-PCR, confirming the importance of the analysis of blood products during DF epidemics. Phylogenetic analysis of partial sequences of 17 samples determined DENV 3 in this first DF outbreak in Cape Verde (Ousmane Faye and Oumar Faye, unpublished data), suggesting importation of DENV by population movements into and between the island. This was confirmed by others [37] and highlighted the need for monitoring and research on DF in West Africa.

### Rift Valley Fever Outbreak 2010, Mauritania

As part of a collaboration between the National Hospital of Nouakchott and the IPD, sera from camels and human cases developing hemorrhagic signs were received between November and December 2010. Rift Valley fever virus was detected in 6 of 7 human and 3 of 5 camel samples by qRT-PCR. After a request from health authorities, a mission was conducted in Mauritania by the IPD from December 8 to 18, 2010 using ML1. A total of 80 samples were collected by the surveillance and investigation teams in Atar, Aoujeft, Akjoujt, Chingetti, Kobeni, and Tintane Moughatas, and qRT-PCR scored 22 of 80 (27.5%) sera and 4 of 85 (4.7%) mosquito pools (2736 mosquitoes) RVFV positive.

At the IPD, 13 sera (59.1%) were positive in IgM-ELISA, and virus was isolated from 6 sera (27.2%) by mouse brain inoculation and mosquito cell culture. Virus was isolated from all 4 RVFV-positive mosquito batches. Phylogenetic analysis of partial L-, S-, and M-segment sequences showed that the strains responsible for the outbreak of RVF re-emerged from a local RVFV focus, showing the need for continuous surveillance [34].

### Yellow Fever Outbreak 2010, Uganda

Laboratory testing conducted by Centers for Disease Control and Prevention (CDC)-Fort Collins since November 2010 confirmed 4 positive cases of YF by PCR and immunohistochemistry in northern Uganda on December 23, 2010. The government of Uganda and WHO/Regional Office of Africa invited the IPD to implement virological and entomological investigations of the YF outbreak and to consult on the control of the outbreak. All human sera and mosquito pools (a total of 112 mosquitoes) collected from affected areas in December 2010 and January 2011 by the Uganda Virus Research Institute/CDC laboratories in Uganda tested negative for YFV and DENV by qRT-PCR. In January 2011, 110 serum samples were collected from patients in 10 districts (Kitgum [15], Agago [25], Kabong [9], Pader [6], Gulu [3], Lamwo [3], Lira [3], Nebbi [2], Yumbe [2], Kitgum [1]). Given the insufficient volume of 68 samples, only 42 were tested and scored negative for YFV and DENV RNA. Additional testing performed in Dakar for IgM antibodies against YFV, DENV, WNV, RVFV, CCHFV, and CHIKV revealed the presence of YF IgM in 8 of 42 samples.

### Ebola Virus Disease Outbreak in Guinea, 2014–2015

The IPD deployed to Conakry on March 23, 2014. The ML2 using qRT-PCR was deployed to the Projet Fièvres Hémorragiques Guinée laboratory at the Donka Hospital. The turnaround time from sampling to result for suspect cases was approximately 3 hours. The ML2 used a 3-station workflow [38].

An increasing number of cases, mainly due to nonreporting, reticence, and infection transmission chains during burials, developed towards the end of 2014. In this context, ML4a/4b were deployed to the periphery of Conakry in 2015, to provide quick results for the safe and dignified burial (SDB) program.

They were setup at Matoto Gbessia Port II district at 2 sites. Positive results were obtained within 30–40 minutes and reported directly to the SDB teams [22].

The ML2 identified (1) the first EVD case in Guinea onsite in Conakry on March 23, 2014 and (2) the first cases of EVD in Liberia from 2 of 7 EBOV-positive samples collected between March 20 and 26, 2014. In total, 1157 of 6055 samples analyzed until May 31, 2015 were EBOV positive. Clinical specimen mainly included serum and swabs samples, and the remainder consisted of urine, amniotic liquid, and placenta fluid (Faye Oumar, Diallo Amadou, Barre Soropogui, Fall Gamou, N'Faly Magassouba, Koivogui Lamine, Sakouba Keita, Loucoubar Cheikh, Weidmann Manfred, Sall Amadou, and Faye Ousmane, manuscript in preparation). Analysis of the spatial distribution of suspected EVD cases revealed confirmation of EBOV in 22 of 29 cases managed by SDB teams.

## DISCUSSION

The development of an ML for molecular diagnostics of HFV lead to the first onsite detection of EBOV in the large EDV outbreak in West Africa in Conakry by a Senegalese team from the IPD on March 23, 2014. This report describes the development of the ML and its tools used during the large EVD outbreak in West Africa in 2014–2015.

For decades, response to outbreaks of VHF relied on deployment of teams from outside of Africa. This allowed outbreaks to gather momentum before onsite investigations began. Fortunately, in most cases, the outbreaks happened in remote, rural settings, thus naturally curtailing the size of the outbreaks due to low population density. However, the EVD outbreak in West Africa demonstrated what can happen if a VHF outbreak moves into urban settings where population density provides the potential for an unlimited reproduction rate (R_0_) of an epidemic.

In this project, we sought to enable a local team to respond to outbreaks and to provide up-to-date molecular diagnostics in an equipment setup that allowed rapid transportation on commercial flights. In exercises in 2010 and 2013 in Kedougou, South East Senegal and in outbreak field deployments to Mauritania, Uganda, and the Cape Verde Islands, the feasibility of the concept was tested. These exercises and deployments helped to improve the ML, which progressively used fewer devices and pipettes and required less energy moving from qRT-PCT to RT-RPA (Table 2).

The molecular assay progressed from using aliquoted PCR primers and probes (ML1) to dried primer and probe reaction mixes (PCR [ML2], RPA [ML3]) to RPA primer in pellets (ML4). The move from PCR to RPA introduced independence from the cooling chain, improved time to result, and led to the development of a suitcase laboratory (DiaS), which was successfully used during the EVD outbreak in Conakry in 2015 [22, 24]. Polymerase amplification in the DiaS has greatly contributed to meeting almost all criteria of the ASSURED criteria [39, 40]. The sensitivity and specificity of RPA equals that of qRT-PCR [22, 41, 42], local teams were trained in the use of ML1-4, and ML4 was used by Guinean teams in Conakry. During the deployment of ML4a/4b, results were usually provided between 30 and 40 minutes after receipt of the sample [22].

The ML4a/b setup is of course still not equipment free. An ML using a SmartCylers costs approximately $100 000 [20]. One fully equipped DiaS costs approximately $9400 and $14 500 with the glovebox. Therefore, by meeting most of the requirements for improved diagnostic tools set by Global Outbreak Alert and Response Network (GOARN) and the WHO Emerging and Dangerous Pathogens Laboratory Network (EDPLN) during the outbreak [43], ML4a/b has the potential to be used widely by African teams as a much more economic option than the tent laboratories that were deployed to West Africa from 2014 to 2015 [7]. Currently, 5 ML4s are based in Senegal, Guinea, Egypt, and Sudan.

Costs for qRT-PCR and RT-RPA including extraction amounted to $8–$10 per reaction, which is not close to the $1 per test target suggested for fieldable point-of-care tests. Intensive research and development for molecular diagnostic point-of-care tests in the last decade has yielded several concepts that have been brought to market or are close to market in industrial countries. The term point-of-care has been used profusely, but the various national health systems in Europe and their reimbursement modalities only allow use of these systems in centralized (high-throughput) laboratories to cover rare diagnostic queries or to cover weekend diagnostic needs at University hospitals [44]. In addition, selling prices at $120/test (FilmArray; BioFire) pose challenges to marketing [45]. During the EDV epidemic in West Africa, the GeneExpert system (qRT-PCR) and the BioFire systems (nested PCR) were evaluated [46, 47]. These systems require high investment in devices, and prices per reaction are not sustainable outside of international funding agencies support. The arrival of the first point-of-care devices should be seen as a positive trend; however, affordable testing will remain an issue for their development and implementation. In the face of these current point-of-care test developments, the DiaS represents a pragmatic approach to taking the current state-of-the-art molecular detection assay into the field without the need for a laboratory tent concept.

The use of the mobile hard plastic glovebox without negative pressure allowed biosafe handling as well as inactivation and extraction of viral RNA. A detailed standard operation procedure was developed and successfully used during the deployment in Guinea. The use of 1% Incidin instead of bleach allowed us to avoid problems caused by the corrosive nature of bleach, which were previously recorded during the EDV laboratory response in West Africa [48].

The future development of this mobile concept will attempt to reduce equipment costs even more by adopting simpler plastic ware concepts and detection devices or paper detection formats. The currently existing DiaS has also been adopted for detection of arboviruses [30, 49] and *Leishmania donovani* [50].

## CONCLUSIONS

In this study, we have shown that it is possible to reduce the amount of equipment and reagents to develop an affordable ML concept to take molecular amplification into the field to deliver diagnostics in outbreak or surveillance settings.

## Notes

***Finanical support.*** This work was funded by European Union grant FP6-INCO-032180, “Development of rapid field diagnostics for identification, control and management of hemorrhagic fever outbreaks” (VHF Diagnostics); Deutschen Gesellschaft für Internationale Zusammenarbeit GmbH, grant VN 81140270, “Improved epidemic control of viral hemorrhagic fever outbreaks in 5 African countries through training of health workers in safely handling and running a novel diagnostic assay of high quality and easy-to use for the detection of viral hemorrhagic fever” (VHF field-diagnostics) and grant 00230020A0 “Improving Fieldable isothermal nucleic acid detection for highly contagious infectious agents”; and Welcome Trust programme: Research for Health in Humanitarian Crises (R2HC) grant 13376, “Point-of-care diagnostic testing for Ebola virus disease in Ebola treatment centres”. The deployment of mobile laboratory 4 (ML4) to Conakry has received special recognition with “Highly Recommended” at the Times Higher Education Award ceremony in 2016 and was also recognized as part of the UNESCO-Equatorial Guinea International Prize for Research in the Life Sciences (awarded to A. A. S.). The development of a suitcase laboratory (DiaS) is also on display at the Science Museum in London.

***Potential conflicts of interest.*** All authors: No reported conflicts of interest. All authors have submitted the ICMJE Form for Disclosure of Potential Conflicts of Interest.

Presented in part: 2015 European Society for Clinical Virology Meeting, Edinburgh.
